# Informed Attentive Predictors: A Generalisable Architecture for Prior Knowledge-Based Assisted Diagnosis of Cancers

**DOI:** 10.3390/s21196484

**Published:** 2021-09-28

**Authors:** Han Li, Linling Qiu, Meihong Wang

**Affiliations:** School of Informatics, Xiamen University, Xiamen 361001, China; lihan@stu.xmu.edu.cn (H.L.); qiulinling@stu.xmu.edu.cn (L.Q.)

**Keywords:** TCGA, cancer prediction and prognosis, prior knowledge, informed machine learning, assist systems

## Abstract

Due to the high mortality of many cancers and their related diseases, the prediction and prognosis techniques of cancers are being extensively studied to assist doctors in making diagnoses. Many machine-learning-based cancer predictors have been put forward, but many of them have failed to become widely utilised due to some crucial problems. For example, most methods require too much training data, which is not always applicable to institutes, and the complicated genetic mutual effects of cancers are generally ignored in many proposed methods. Moreover, a majority of these assist models are actually not safe to use, as they are generally built on black-box machine learners that lack references from related field knowledge. We observe that few machine-learning-based cancer predictors are capable of employing prior knowledge (PrK) to mitigate these issues. Therefore, in this paper, we propose a generalisable informed machine learning architecture named the Informed Attentive Predictor (IAP) to make PrK available to the predictor’s decision-making phases and apply it to the field of cancer prediction. Specifically, we make several implementations of the IAP and evaluate its performance on six TCGA datasets to demonstrate the effectiveness of our architecture as an assist system framework for actual clinical usage. The experimental results show a noticeable improvement in IAP models on accuracies, f1-scores and recall rates compared to their non-IAP counterparts (i.e., basic predictors).

## 1. Introduction

Cancer is a major cause of human death, as it often leads to a series of related fatal diseases that are complicated to control [1]. As a common sign of cancer, cancer tumours are often caused by different genetic mutations and generally display considerable phenotypic heterogeneity in cancer cells [2]. It is, therefore, a common challenge among researchers to eliminate cancers, because the genetic pathogenic mechanisms of cancer tumours are too complex to be observed or properly understood [3].

Due to its complexity, the clinical treatment of cancer is in high demand, the goal of which is more practical and real-life reasonable. The early detection and prediction techniques of cancers, which facilitate successive medical treatment of patients, are being extensively studied [4] in order to assist doctors in making early diagnoses, thus improving the survival rate of cancer cases and helping discover useful insights into disease pathogenesis [5].

With the improvement of device performance and accuracy, DNA sequencing techniques are now able to collect genomic profiles of tumour samples, such as Gene Expression (GE), miRNA Expression (ME) and DNA Methylation (DM), without extensive physical or mental exertion. While it is demanding to excavate possible patterns of tumour-causing gene expression signatures from clinical data for even well-experienced physicians, many data-driven approaches can be used to assist doctors in the early detection of cancers. Because of their ability to discover feature patterns from large-scale data, machine learning algorithms such as Support Vector Machines (SVMs), Bayesian Networks (BNs) and Decision Trees (DTs) have been well employed for modelling the development and treatment of cancer conditions [6,7,8]. These methods are useful for early cancer detection, as they can automatically capture the effective patterns of samples, which are more likely to be diagnosed as tumour tissue.

Many machine learning methods use gene expression signatures as inputs, which indicates a distinctive, species- or tissue-specific expression pattern of genes in one cell [9]. While it is true that gene signatures could considerably enhance the capabilities of cancer prognosis, many relevant methods are not suitable for medical practice, as they lack necessary intelligent frameworks when facing the large, complex features of real-world cancer data [10]. Hence, deep learning techniques such as Artificial Neural Networks (ANNs), Recurrent Neural Networks (RNNs) and Deep Neural Networks (DNNs) are integrated to improve the abstractness of cancer prediction and prognosis models, resulting in lower training time, better feature extraction quality and enhanced diagnosis accuracy compared to machine learning techniques [11].

There are a series of deep learning models that achieve elevated performance on certain cancer prediction tasks [12,13,14]. However, the structure of many deep networks is too complex to be understood, as they are often composed of multiple deep layers where high-level, abstract-entity representations are transformed in between. In addition, the gene signature of every sample can vary to a huge extent. A complex, nonlinear and arbitrary decision boundary is often associated with cancer diagnosis [15]. Thus, to mitigate the possibility of overfitting, a large learning dataset is often of necessity for the training process of deep learners [13], which is not necessarily available for certain diseases, and may lead to common data-driven failures such as gradient vanishing [16,17].

Recently, the notion of IML has gained a growing interest, which is especially considered a potential solution for dealing with insufficient training data [18]. Unlike traditional approaches, where knowledge is generally used for preprocessing or feature engineering on training data, IML defines a process through which the model could learn from a composite of multiple information sources that consist of both data and prior knowledge (PrK); the latter is explicitly integrated into the machine learning pipeline from an independent knowledge source [18]. By integrating human experience, e.g., equations, probabilities and knowledge graphs, as PrK into the learning process, the reliability and robustness of the underlying model can be improved [19].

According to our knowledge, few methods in relevant fields are already able to integrate the novel concept of IML into the cancer prediction task, and therefore, few approaches are capable of leveraging PrK to assist cancer diagnosis. Inspired by this, we designed and proposed a conceptual architecture, the Informed Attentive Predictor (IAP), which aims at the cancer prediction task, as well as its extended field application, namely assisted diagnosis of cancers. Moreover, the IAP embodies the idea of informed machine learning (IML) and is designed to be generalisable, i.e., it describes a method of knowledge integration where the human experience straightforwardly takes part in the decision-making process, which is classifier-independent. Several IAP implementations were constructed and evaluated to demonstrate their effectiveness on either full- or reduced-size datasets.

To summarise, our main contributions are threefold:

1. We propose an end-to-end IML architecture (IAP) for the assisted diagnosis of cancers, which describes a method through which the underlying classifier can fuse human experience into its whole process, even if the training data have a limited size. Because the PrK integration module is dependent on the underlying classifier, the IAP is totally generalisable. To the best of our knowledge, the IAP is the first attempt to incorporate IML with cancer prediction.
2. Based on some previously proposed classifiers targeted at the same task, we developed and evaluated several IAP implementations. Specifically, we compared the performance between each IAP implementation and its basic counterpart. Experiments show that by applying the IAP architecture, model performance is improved by a noticeable margin on either full- or reduced-size datasets.
3. The comparative analyses amongst multiple experiments show that our proposed architecture and models achieve higher accuracies, F1-scores and recall on the tested TCGA datasets. Thus, the validity and performance of our methodologies are verified.

## 2. Related Work

In this section, we exemplify related work targeting the cancer prediction task. Specifically, we focus on methods with machine learning and/or deep learning techniques, as well as some recent applications of IML in relevant fields.

### 2.1. Machine Learning Methods for Cancer Prediction

Machine Learning (ML) is a branch of Artificial Intelligence (AI) research that utilises a variety of statistical and probabilistic techniques to automatically learn patterns of features from past examples (train) and then use the trained model to classify new data [20].

Traditional Artificial Neural Networks (ANNs) [21], Support Vector Machines (SVMs) [22] and Decision Trees (DTs) [23] have been widely used in cancer detection and diagnosis for approximately two decades [24,25,26]. In [27,28,29], different types of ANNs are employed to build effective cancer prediction models to learn from cell characteristics and gene subsets. The authors of [30,31,32] utilise microarray gene expression data to train their models in cancer prediction and prognosis using ANN algorithms. Refs. [33,34,35,36] presents examples of SVMs used in cancer prediction and prognosis. Apart from methods that employ only one ML technique, many articles, including [37,38], propose synthesis models that employ more than two ML algorithms for cancer prediction.

### 2.2. Deep Learning Methods for Cancer Prediction

With the development of computational devices, Deep Learning (DL) methods started to play an important role in the construction of classifiers. A DL model has a branch of multiple processing layers, through which representations of data can be learnt in a way that contains different levels of abstraction [39].

A majority of DL methods utilise the Convolution Neural Network (CNN), a feed-forward network that allows for effective feature extraction by applying convolution on embeddings. For instance, in [40], the authors propose a model where multiple CNN-based architectures are implemented and employed to distinguish cancer of different types based on a heterogeneous dataset. In addition, CNN-based models were and are being used in a wide range of cancer prediction tasks, including those that employ Computer Vision (CV) techniques to detect and classify tumour types based on medical images [41,42]. Recently, some newly developed CNN-based DL algorithms have been used in cancer detection and prediction. In [36], a deep feature approach, with a GoogleNet model pretrained on ImageNet used as the feature extractor and a polynomial kernel SVM used as the classifier, was used to identify occult invasive disease in patients diagnosed with Ductal Carcinoma in Situ (DCiS) by core needle biopsy.

However, [43] mentioned that several problems arise from the existing deep learning models, such as overfitting, bad performance with small datasets and the struggle to deal with noisy features. Therefore, several techniques that make use of novel fields of deep learning have been proposed to handle some of these problems, such as BDR-CNN-GCN [44], which incorporates Graph Convolution Network (GCN) and CNN; some newly developed deep neural networks, as discussed by the authors of [45], who use Graph Attention Network (GAT) to identify personalised prognostic markers; and Gated Graph Attention (GGAT) network [46], which uses a gating mechanism to enhance its underlying GAT classifier.

### 2.3. Relevant Development of Informed Machine Learning

As research into cancers has progressed, multiple large-scale datasets have been made available. However, the heterogeneity of these datasets makes it difficult for researchers to construct cancer predictors that learn from multiple data sources. In addition, DL models are becoming much less explainable, as multiple layers have to be stuck to abstract the representations of numerous features.

As a source of information, PrK has been used to enhance the performance of many ML and DL classifiers. The ML techniques that learn from a synthesis of multiple information sources that consist of data and PrK can be summarised by a recently proposed term, i.e., IML [18]. By employing PrK into the ML pipeline (i.e., training and/or prediction process), fewer data are necessary for learning, and the results of small models can be significantly improved.

IML has been used in a wide range of research fields. In [47], the authors use a modern machine learning technique based on random forests to predict Reynolds stress discrepancies in different flows where data are not available, with existing direct numerical simulation databases used to train discrepancy functions. The authors of [48] propose a methodology for using symbolic knowledge in DL by learning a semantic loss function that bridges between neural output vectors and logical constraints. The authors of [49] propose a constraint-based framework for learning user-specified constraints (e.g., physical laws) and simultaneously using them for supervision. This is useful to mitigate the difficulty of using domain expertise to manually specify constraints.

Furthermore, PrK has also been made use of in many biological and medical fields. In [50], a predictor named immunological Elastic-Net (iEN) is integrated with outside immunological knowledge, resulting in the model demonstrating predictions of higher quality for some clinical outcomes on a dataset of large feature size. In [51], protein–protein interaction knowledge is utilised in their gene selection process. This improves the interpretability of gene signatures compared to pure deep learning models.

As to cancer-related methodologies, the authors of [52] incorporate relevant genetic information extracted from former literature with an SVM classifier that enables the usage of known lung adenocarcinoma genes in its cancer classification task. The accuracy is, therefore, improved due to the more effective method of gene selection.

However, we can find a few additional articles on methods that integrate the concept of IML into the cancer prediction task. Therefore, in this study, we propose the IAP that describes an end-to-end method to incorporate the IML concept with basic classifiers to predict cancers based on gene expression signatures. Unlike the aforementioned PrK-based models, we fuse the knowledge in a different way that aims at enhancing prediction instead of features, which is more generalisable because the underlying classifier can be altered or specifically designed for the targeted task.

## 3. Methodologies

In this section, we describe the formulations of our architecture and the associated task. We initially describe the cancer prediction task that is considered in this work; then, we describe an introduction to the preprocessing of data. Finally, we put forward the structure of our IAP and give a detailed explanation of the methodologies.

### 3.1. Problem Formulations

The cancer prediction task considered in this work is essentially the classification of samples, which takes features (i.e., gene expression) of samples as input and gives predictions (i.e., cancer or not) accordingly. The aim of this task is to build an errorless and robust classifier by which a high classification accuracy can, even on new samples, be obtained.

### 3.2. Terminology

The symbols used in this paper are listed in Table 1. Note that the terms ‘sample’, ‘entity’ and ‘node’ in different contexts refer to the same concept in terms of ‘samples’ in a dataset and ‘entities’ or ‘node’ in graph-structured data.

### 3.3. Data Preprocessing

We follow up several preprocessing steps to extract PrK from its data source. In addition, the original dataset is standardised into graph-structured data to meet the needs of graph-based IAP models.

#### 3.3.1. Construction of Prior Knowledge

As one of our major contributions, we employ the idea of IML in our model by integrating PrK from another data source. The fusing process varies according to different types of PrK and the supporting task. In this work, we use PrK as an aid for the training process in a 4-step manner: (1) PrK extraction, (2) data reduction, (3) PrK encoding and (4) PrK-based difference amplification. The initial two steps, viz. PrK extraction and data reduction, take place in the preprocessing period. The latter two steps are both taken in the training period. We propose their description in later sections.

1. PrK Extraction

The types of available PrK and the corresponding obtaining methods vary for different research contexts, and they require human effort to design the specific procedure for PrK extraction and encoding. For instance, if the training data are served in forms such as WHI images, it would be convenient to employ pathological image patterns of confirmed cases as PrK to enhance the convoluted representations of images. In this work, based on the type of dataset used (i.e., gene expression rate for tumour and normal samples), it is natural to find relevant gene-related knowledge, be it gene mutation rates of the cancers released by a recognized authority, relative expression levels of different genes recorded in an NIH database, etc.

Through careful investigation, we eventually selected mutation rate as the source of PrK, because it fits well to the main procedure of knowledge integration (Section 3.4.2) and is not model specific. We extracted a statistical table reported by a previous study towards the cooccurrence and exclusiveness of gene mutations amongst several cancer types [53], where the mutation rates of more than a hundred genes are recorded in percentages. Then, we pulled out the values of each cancer type (viz. each column) from the table, with each represented as a knowledge vector $k_{c}\in R^{N_{k}}$. They were then put aside for the later steps of training.

2. Data Reduction

Once all $k_{c}$ were obtained, we extracted the expression values corresponding to the reported $N_{k}$ genes from the feature matrix, thereby producing a ‘reduced’ feature vector ${\tilde{e}}_{i}\in R^{N_{k}}$ for each entity *i*. ${\tilde{e}}_{i}$ is formulated in Equation (1)

(1) $$\tilde{e_{i}}={\left[e_{im_{1}},e_{im_{2}},\cdots ,e_{im_{N_{k}}}\right]}^{T}$$

where $e_{im_{g}}$ ($g\in 1,2,\cdots ,N_{k}$) indicates the corresponding feature value (viz. gene expression value) in $e_{i}$ in terms of the *g*-th reported gene. The ‘reduced’ feature vectors of all samples were produced and saved for later utilisation.

The matrices and vectors produced in the preprocessing period, i.e., adjacency matrix A ($A\in R^{N\times N}$), feature matrix $E=\left[e_{1},e_{2},\cdots ,e_{N}\right]$ ($E\in R^{N_{e}\times N}$), knowledge vectors and reduced feature vectors, are inputs of our proposed model.

#### 3.3.2. Graph Construction

We standardised the original datasets into undirected and unweighted graphs, as some graph-based classifiers (e.g., GAT, GCN, GGAT) take graph-structured data as their input. For simplicity, we constructed the graph according to the vector distances between the original samples. Specifically, we assumed two samples to be ‘correlated’ if their vector distance equalled or was smaller than the threshold. We used cosine similarity as the metric of the distance between entities, as described in Equation (2).

(2) $$l_{ij}=\frac{e_{i}\cdot e_{j}}{∥e_{i}∥∥e_{j}∥}=\frac{\sum_{m=1}^{N_{e}}e_{im}e_{jm}}{\sqrt{\sum_{m=1}^{N_{e}}e_{im}^{2}}\sqrt{\sum_{m=1}^{N_{e}}e_{jm}^{2}}}$$

The adjacency matrix of the graph was then constructed according to Equation (3)

(3) $$A_{ij}=\left\{\begin{array}{cc} 1 & \mathrm{if}\;l_{ij}\leq \theta_{i},\\ 0 & \mathrm{otherwise} \end{array}\right.$$

where $\theta_{i}$ is the threshold value for entity *i*. We used the mean distance of all neighbours as the threshold, i.e.,

(4) $$\theta_{i}=\left.\sum_{j\in N_{i}}l_{ij}/|N_{i}|\right.$$

### 3.4. IAP Structure and Procedure

**Figure 1:**
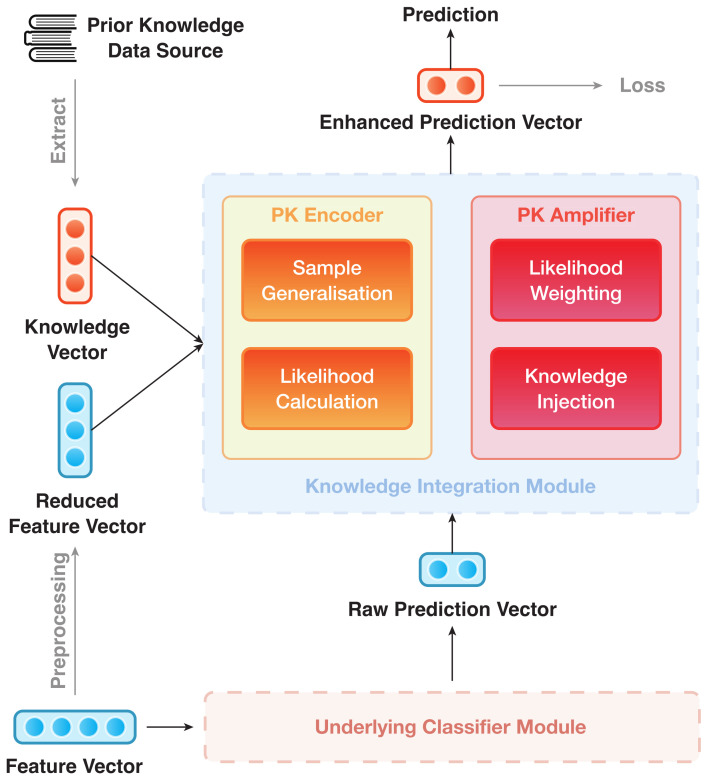
Overall structure of IAP.

**Figure 2:**
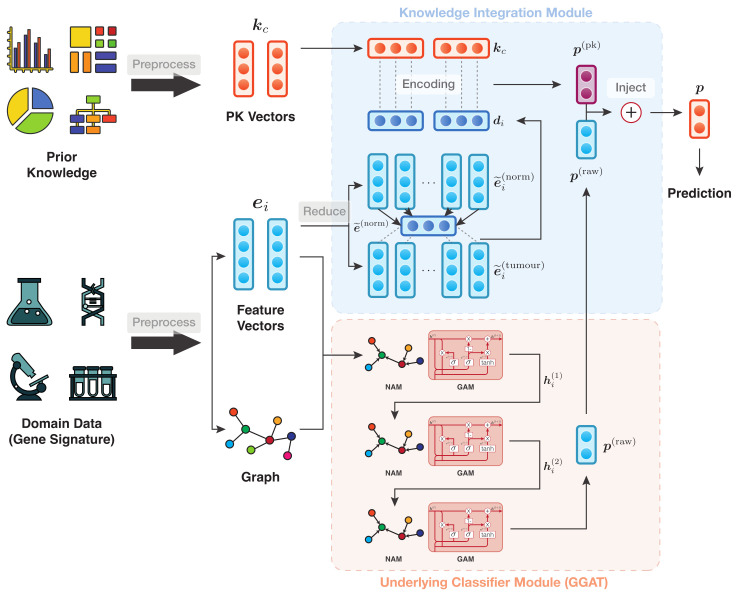
Data flow of IAP-GGAT.

The IAP architecture can be divided into modules, viz. (1) Underlying Classifier Module (UCM) and (2) Knowledge Integration Module (KIM). They are trained in an end-to-end manner. The overall structure of IAP is shown in Figure 1, where the UCM is rendered as a black box. As an example, Figure 2 depicts the data flow of an IAP implementation of GGAT [46] (IAP-GGAT).

The subsequent part will follow up the division in Figure 1 and give details of the two modules and their submodules accordingly.

#### 3.4.1. Underlying Classifier Module

The UCM aims at giving a basic prediction result based on the original dataset. The prediction vectors are then fed into the KIM and enhanced therein. The enhancements will be formalised in detail in Section 3.4.2. Here, we treat the UCM as a black box that takes feature vectors as input and produces raw prediction vectors ($p_{i}^{\left(\mathrm{raw}\right)}\in R^{2}$). The overall procedure of the UCM can be formalised as a function $P^{\left(\mathrm{raw}\right)}=\mathrm{UCM}\left(E\right)$, where $P^{\left(\mathrm{raw}\right)}=\left[p_{1}^{\left(\mathrm{raw}\right)},p_{2}^{\left(\mathrm{raw}\right)},\cdots p_{N}^{\left(\mathrm{raw}\right)}\right]$ indicates the raw predictions set against all samples. The function $\mathrm{UCM}\left(\cdot \right)$ indicates the abstract procedure of the UCM. For graph-based UCMs, the adjacency matrix is taken into consideration, resulting in the graph-based UCM overall procedure that is formalised as the function $P^{\left(\mathrm{raw}\right)}=\mathrm{UCM}\left(E,A\right)$.

To be precise, $P^{\left(\mathrm{raw}\right)}$ contains logits of the classifier from which predictions can directly be derived. To improve their quality, the logits are enhanced by an ‘injection’ of PrK during the procedure of KIM.

#### 3.4.2. Knowledge Integration Module

As a key feature of IML, the KIM serves as an extension to the UCM. It learns an effective way to fuse PrK into the prediction process (that is, to take the 3rd and 4th steps of the IAP mentioned in Section 3.3.1). These two steps are performed accordingly by the two key parts of the module: (1) PrK Encoder and (2) PrK Amplifier.

**PrK Encoder** is used to map both PrK and reduced data into a uniformed vector space and therein produce a separate prediction that depends solely on the PrK. This simple prediction is not intended for practical use. We only utilise the prediction vectors to amplify and enhance the logits from the UCM.

The PrK encoder follows a 2-step procedure: sample generalisation and likelihood calculation. In the ‘sample generalisation’ period, PrK Encoder calculates a general representation for *normal* samples by applying a linear transform to the ‘reduced’ feature vectors, as indicated by Equation (5)

(5) $${\tilde{e}}^{\left(\mathrm{norm}\right)}=\sum_{i=1}^{\left|V^{\left(\mathrm{norm}\right)}\right|}v_{i}{\tilde{e}}_{V_{i}^{\left(\mathrm{norm}\right)}}={\tilde{E}}^{\left(\mathrm{norm}\right)}v$$

where $V^{\left(\mathrm{norm}\right)}$ indicates a set that consists of the reference numbers of normal samples, and $V_{i}^{\left(\mathrm{norm}\right)}$ is the *i*-th element of $V^{\left(\mathrm{norm}\right)}$. ${\tilde{E}}^{\left(\mathrm{norm}\right)}\in R^{N_{k}\times \left|V^{\left(\mathrm{norm}\right)}\right|}$ is a reduced feature matrix of normal samples, and $v\in R^{\left|V^{\left(\mathrm{norm}\right)}\right|}$ is a projection vector that squashes ${\tilde{E}}^{\left(\mathrm{norm}\right)}$ into a one-column matrix, i.e., the general representation for normal samples (${\tilde{e}}^{\left(\mathrm{norm}\right)}\in R^{N_{k}}$).

${\tilde{e}}^{\left(\mathrm{norm}\right)}$ can be seen as a learnt representative of normal samples, as the projection vector v can be optimised as training progresses.

In the ‘likelihood calculation’ period, PrK Encoder measures the exact difference between the reduced feature vector of each sample (regardless of its actual type, i.e., normal or tumour) and the normal sample representative, which is described as Equation (6).

(6) $$d_{i}=\frac{\left|{\tilde{e}}^{\left(\mathrm{norm}\right)}-{\tilde{e}}_{i}\right|}{{\tilde{e}}^{\left(\mathrm{norm}\right)}}$$

In $d_{i}\in R^{N_{k}}$, each component indicates the relative difference of the corresponding gene expression value with respect to its counterpart in the normal sample representative. Intuitively, $d_{i}$ measures the extent to which *i* and normal samples are dissimilar in terms of gene expression, and so does the gene mutation rate. Inspired by this, a value of how likely this sample can be categorised as tumour type *c* can be obtained by calculating an element-wise similarity between $d_{i}$ and $k_{c}$, as described in Equation (7)

(7) $$\begin{array}{cc} s_{ic} & =\left|\mathrm{sim}\left(d_{i},k_{c}\right)\right|\\ & =\left|\mathrm{sim}\left(\frac{\left|{\tilde{e}}^{\left(\mathrm{norm}\right)}-{\tilde{e}}_{i}\right|}{{\tilde{e}}^{\left(\mathrm{norm}\right)}},k_{c}\right)\right| \end{array}$$

where the function $\mathrm{sim}\left(a,b\right)$ is used to calculate the similarity between two vectors, a and b. Here, considering that the function takes two vectors ($d_{i}$ and $k_{c}$) of different semantic meanings, we employ cosine similarity, which is not sensitive to the exact components values being compared, to mitigate the potential influence of the mismatched magnitudes between them. The output, $s_{ic}\in \left(0,1\right)$, is a scalar, indicating the *likelihood* of sample *i* being diagnosed as tumour type *c* with only a reference to PrK. A prediction vector can thereby be constructed according to Equation (8)

(8) $$p_{i}^{\left(\mathrm{pk}\right)}={\left[1-s_{ic},s_{ic}\right]}^{T}$$

with the first and second components of $p_{i}^{\left(\mathrm{pk}\right)}\in R^{2}$ indicating the prediction of sample *i* being a normal and tumour sample, respectively. This prediction vector, as previously mentioned, is not intended to make any final prediction or diagnosis. It is only a by-product that will then be further utilised in PrK Amplifier.

**PrK Amplifier** uses $p_{i}^{\left(\mathrm{pk}\right)}$ to amplify and enhance the results of raw prediction vectors and was previously introduced in Section 3.4.1. The procedure of PrK Amplifier can, likewise, be broken down into 2 simple steps: likelihood weighting and knowledge injection.

In the ‘likelihood weighting’ phase, a weighted version of each $p_{i}^{\left(\mathrm{pk}\right)}$ is obtained, ($p_{i}^{\left(\mathrm{weighted}\right)}$), by assigning different weights to the prediction values. Each $p_{i}^{\left(\mathrm{weighted}\right)}$ is then ‘injected’ into the raw prediction vector accordingly by a simple summation in the ‘knowledge injection’ phase. This action will eventually result in an amplification or weakening of either component in the prediction vector.

The two steps of PrK Amplifier can be jointly formalised as Equation (9)

(9) $$p_{i}=\mathrm{softmax}\left(p_{i}^{\left(\mathrm{raw}\right)}+w⊙p_{i}^{\left(\mathrm{pk}\right)}\right)$$

where $w\in R^{2}$ is optimised as the training progresses. Being normalised by the softmax function, the resulting $p_{i}$ is the ‘adjusted’ prediction vector whose first and second components indicate the final prediction of sample *i* being a normal and a tumour sample, respectively.

$p_{i}$ is also the output of IAP, on which the optimiser calculates loss and performs fine-tuning on parameters through gradient descending.

#### 3.4.3. Example of IAP Implementation

As an example, Figure 2 depicts the data flow of an IAP implementation of GGAT [46] (IAP-GGAT).

In this example, the domain data, i.e., gene signature of cancer/normal samples, are preprocessed to a graph-like structure. The graph is then fed into the first GGAT layer of the UIM, followed by two subsequent processes performed by the latter two GGAT layers. Furthermore, as in KIM, the reduced feature vectors are squashed into one, which is then used by the encoder to give predictions based on knowledge and features. The prediction vectors produced, i.e., $p^{\left(\mathrm{pk}\right)}$, are ‘injected’ into the logits of the final GGAT layer, i.e., $p^{\left(\mathrm{raw}\right)}$, through a procedure that we call ‘knowledge injection’. The injected prediction vectors are the final results of the whole model and will finally be used in calculating the loss function and model optimisation.

## 4. Evaluation

We implemented four IAP models based on two traditional machine learning classifiers that were previously utilised in similar cancer prediction tasks [35,54], i.e., Support Vector Machine (SVM) and Decision Tree (DT), and two newly developed graph-based deep learning classifiers, i.e., Graph Attention Network (GAT) and Gated Graph Attention Network (GGAT). These models are identified by their original names plus a prefix ‘IAP-’, i.e., IAP-SVM, IAP-DT, IAP-GAT and IAP-GGAT, respectively.

To evaluate the effectiveness of the IAP, we carried out cancer prediction experiments on several TCGA datasets that are available to the public. All of these datasets were initially preprocessed (Section 3.3) into graph-like versions for the four graph-based candidate models (GAT, IAP-GAT, GGAT, IAP-GGAT). Furthermore, we extracted the PrK from [53] and constructed it into specific formats. All experiments were performed in a unified environment, where PyTorch [55] was used to implement our models. In addition to normal experiments, we give details on a supportive experiment that was intended for demonstrating the effectiveness of IAP with the training data size being reduced.

### 4.1. Introduction and Modifications to Datasets

The datasets on which we carried out experiments were released by The Cancer Genome Atlas (TCGA), a cancer genomics programme of the U.S. National Cancer Institute and the U.S. National Human Genome Research Institute, each containing gene expression values (gene signatures) of samples from either a specific type of tumour or normal tissues. Detailed information on TCGA can be found in https://www.cancer.gov/about-nci/organization/ccg/research/structural-genomics/tcga (accessed on 28 September 2021). The datasets can be downloaded from the Genomic Data Commons (GDC) Data Portal (https://portal.gdc.cancer.gov/ (accessed on 28 September 2021)) of the U.S. National Institutes of Health (NIH).

#### 4.1.1. Statistics and Visualisation of Datasets

In this work, we chose six datasets (tumour types) for evaluation, because they are covered by both TCGA and the original research article of the PrK: Urothelial Bladder Carcinoma (BLCA), Breast-Invasive Carcinoma (BRCA), Head and Neck Squamous Cell Carcinoma (HNSC), Kidney Renal Clear Cell Carcinoma (KIRC), Lung Adenocarcinoma (LUAD) and Lung Squamous Cell Carcinoma (LUSC). Statistics of these datasets are available in Table 2.

Here, we utilise t-SNE [56] to visualise the samples in these datasets and coloured the nodes based on their type (Tumour or Normal).

#### 4.1.2. Data Balancing

We observed an imbalance between the two tumour types in terms of the number of samples, i.e., the number of tumour samples is much more than that of the normal samples in any dataset. This disparity can cause a variety of problems, such as overclassifying the majority group [57] and slow convergence of the neural network [58]. Several solutions can be applied to couple this issue, including data-level methods such as Random Undersampling (RUS), Random Oversampling (ROS) [59], SMOTE [60] and ADASYN [61], as well as algorithm-level methods such as cost-sensitive learning, which assigns penalties into different classes [62].

**Figure 3:**
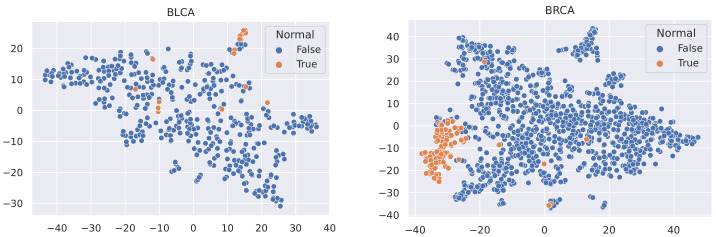
t-SNE visualisation of BLCA and BRCA.

**Figure 4:**
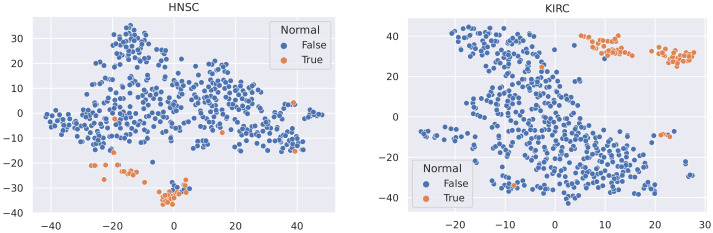
t-SNE visualisation of HNSC and KIRC.

**Figure 5:**
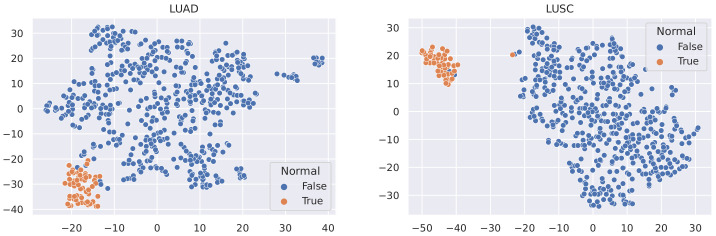
t-SNE visualisation of LUAD and LUSC.

It is not reasonable to mitigate the number of any sample types, as the datasets used are all small in size (no more than 1215 samples). In this case, to minimise the effect of data imbalance, we only consider methods that increase the number of minority samples. Advanced upsampling techniques such as ADASYN and SMOTE are generative (that is, they produce new samples according to certain statistical rules). However, the generation of these artificial samples does not involve specialised knowledge. They simply use Euclidean distance and random bias to sample new data. Furthermore, according to the t-SNE visualisation of the datasets (Figure 3, Figure 4 and Figure 5), it can be observed that the areas of the two classes slightly overlap with each other. For some datasets such as BLCA, the two classes are actually not spatially divisible. We believe the main reason for this mismatch is the complex co-relation within gene sequences. This means that these generated samples may be misleading because their spatial location is not necessarily related to the actual classes. Therefore, we applied ROS on the minority class (i.e., normal samples), i.e., we randomly duplicated the normal samples to make sure that the two classes were equal in size when training the models (i.e., Normal and Tumour).

### 4.2. Experiments

In our experiments, the parameters of our model are initialised using Xavier initialization [63] and are then trained using the Adam optimiser [64] with an initial learning rate set to $5\times 10^{-3}$. Graph-based models (GAT, GGAT) are trained with 1500 epochs, whilst for others, the number of epochs is set to 150. We report each candidate model’s classification performance, measured by a number of different metrics, on each dataset.

To mitigate the occurrence of possible exceptions, we employed k-fold cross-validation in our experiments, where the datasets are randomly divided into five parts, and each is then utilised to test the models. Eventually, the average performance of the five test sets is reported. Considering the size of the datasets, we set k = 5 to ensure that the test sets were not too small whilst enabling adequate repetitive experiments.

#### 4.2.1. Training Process

**Figure 6:**
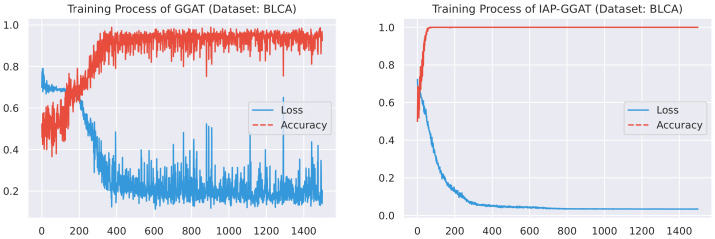
Training process of GGAT and IAP-GGAT on BLCA dataset.

**Figure 7:**
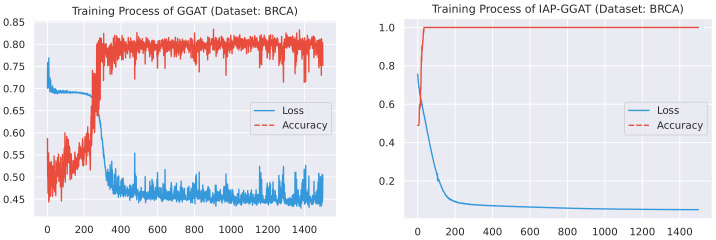
Training process of GGAT and IAP-GGAT on BRCA dataset.

**Figure 8:**
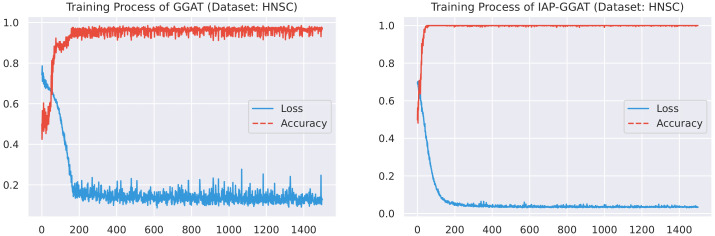
Training process of GGAT and IAP-GGAT on HNSC dataset.

**Figure 9:**
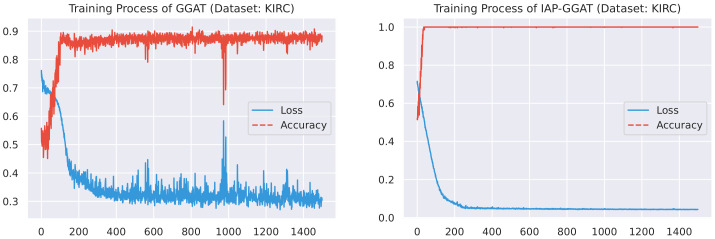
Training process of GGAT and IAP-GGAT on KIRC dataset.

**Figure 10:**
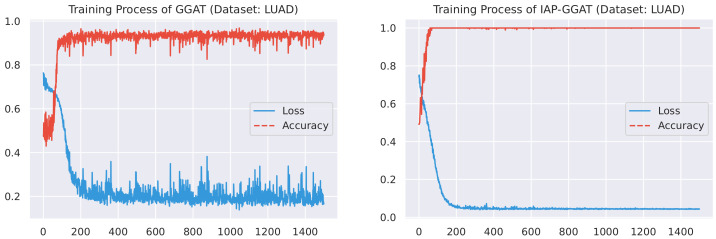
Training process of GGAT and IAP-GGAT on LUAD dataset.

**Figure 11:**
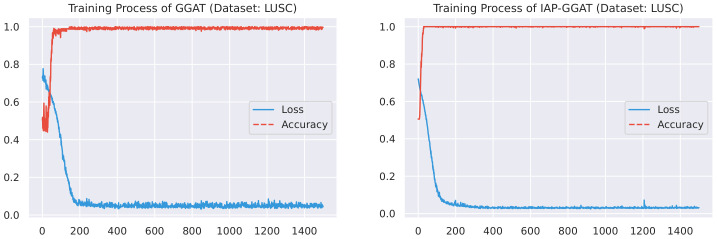
Training process of GGAT and IAP-GGAT on LUSC dataset.

Here, we plot the changes of accuracies and loss values during GGAT’s and IAP-GGAT’s training process in Figure 6, Figure 7, Figure 8, Figure 9, Figure 10 and Figure 11 to study the learning process of IAP (first 1500 epochs). As a comparison, we also plot the same figures for both GAT and IAP-GAT in Appendix A on which the same conclusions can be derived.

Noticeably, for IAP-GGAT, the learning curve has a highly remarkable smoothness, whereas there is observable volatility of GGAT when reaching its plateau. Under extreme circumstances, as in KIRC, for example, where a sudden expansion of loss occurs at approximately the 980th epoch, we can hardly observe even marginal changes in either loss or accuracy of IAP-GGAT at the corresponding moment. Furthermore, the time needed for IAP-GGAT to converge (all less than 100 epochs) is visibly shorter than that for GGAT (between 50 and 400 epochs). These differences highlight two strengths of IAP models: (1) less volatile training curve and (2) less time required for model convergence.

#### 4.2.2. Experimental Results

The experimental results in terms of accuracy, recall and F1-score are shown in Table 3, Table 4 and Table 5, where:

1. The best results are in **bold texts**;
2. A number marked ‘+’ indicates a higher result in the corresponding pair of models;
3. A number marked ‘=’ indicates that the results of corresponding pair of models are equal to each other;
4. The values in the rightmost column are the count of ‘+’s that the corresponding models achieve on the datasets listed.

The experimental results show that all of our IAP implementations, on average, achieved better performance on a majority of datasets compared to their non-IAP counterparts. Only a marginal difference can be observed where the IAP models ranked second. Furthermore, in most cases, the IAP models achieve more ‘+’s than their basic version, with the exception of recall on (IAP-)SVM, where small differences can be discovered in the experimental results on BRCA and KIRC datasets. We believe the occurrence of this is occasional and can be amended by refining the model’s initial settings (e.g., SVM’s kernel type). Nevertheless, the effectiveness of our PrK integration technique is clearly highlighted.

**Table 3:** Accuracy on all datasets. (†) PrK-ONLY: a naïve model that only facilitates the PrK for prediction.

Models	BLCA	BRCA	HNSC	KIRC	LUAD	LUSC	Avg.	+
DT	0.9880	0.9943	0.9925	0.9768	0.9906	0.9950+	0.9895	(1+)
IAP-DT	0.9940+	0.9943=	0.9925=	0.9953+	0.9953+	0.9925	0.9940+	(4+)
SVM	0.9910	0.9943	0.9875	0.9861+	0.9906	0.9975	0.9912	(1+)
IAP-SVM	0.9970+	0.9943=	0.9925+	0.9860	0.9953+	**1.0000**+	0.9934+	(5+)
GAT	0.9578	0.8367	0.9800	0.9070	0.9343	0.9975	0.9356	(0+)
IAP-GAT	0.9910+	0.9864+	0.9950+	0.9837+	0.9812+	**1.0000**+	0.9896+	(7+)
GGAT	0.8589	0.6039	0.9980	0.9935	0.9953	0.9990	0.9081	(0+)
IAP-GGAT	**1.0000**+	**0.9977**+	**0.9990**+	**0.9935**=	**0.9981**+	0.9990=	**0.9979**+	(5+)
PrK-ONLY†	0.4880	0.5102	0.4700	0.5278	0.5234	0.4726	0.4987	N/A

**Table 4:** Recall on all datasets.

Models	BLCA	BRCA	HNSC	KIRC	LUAD	LUSC	Avg.	+
DT	0.9781	0.9891+	0.9798	0.9592	0.9986	0.9932	0.9830	(1+)
IAP-DT	0.9867+	0.9884	0.9798=	0.9907+	0.9986=	0.9932=	0.9896+	(3+)
SVM	0.9833	0.9897+	0.9943	0.9860+	0.9954	0.9955	0.9907+	(3+)
IAP-SVM	0.9940+	0.9884	0.9943=	0.9695	0.9954=	**1.0000**+	0.9903	(2+)
GAT	0.9432	0.8345	0.9798	0.9292	0.9095	0.9957	0.9320	(0+)
IAP-GAT	**1.0000**+	0.9879+	0.9923+	0.9986+	0.9953+	**1.0000**+	0.9957+	(7+)
GGAT	0.8950	0.7392	0.9961	0.9871	0.9963	0.9982	0.9353	(0+)
IAP-GGAT	**1.0000**+	**0.9932**+	**1.0000**+	**0.9987**+	**1.0000**+	**1.0000**+	**0.9987**+	(7+)

**Table 5:** F1-score on all datasets.

Models	BLCA	BRCA	HNSC	KIRC	LUAD	LUSC	Avg.	+
DT	0.9889	0.9945+	0.9927+	0.9770	0.9908	0.9951+	0.9898	(3+)
IAP-DT	0.9932+	0.9942	0.9916	0.9953+	0.9952+	0.9924	0.9937+	(4+)
SVM	0.9915	0.9948+	0.9882	0.9860+	0.9909	0.9977	0.9915	(2+)
IAP-SVM	0.9970+	0.9942	0.9917+	0.9844	0.9953+	**1.0000**+	0.9938+	(5+)
GAT	0.9551	0.8451	0.9798	0.9079	0.9310	0.9973	0.9360	(0+)
IAP-GAT	0.9908+	0.9862+	0.9950+	0.9846+	0.9804+	**1.0000**+	0.9895+	(7+)
GGAT	0.8904	0.5997	0.9980	0.9934	0.9952	0.9989	0.9126	(0+)
IAP-GGAT	**1.0000**+	**0.9978**+	**0.9990**+	**0.9935**+	**0.9981**+	0.9991+	**0.9979**+	(7+)

Additionally, for ablation study purposes, we introduce a control group of experiments performed on a naïve classification model that makes decisions based only on PrK (without other underlying models), whose performance in terms of accuracy is shown as ‘PrK-ONLY’ in Table 3. The PrK-ONLY model utilises $p^{\left(\mathrm{pk}\right)}$ for direct classification. According to our aforementioned discussion, the prediction vectors produced by the PrK integration module are only intended to be used to enhance the prediction of the underlying classification model. In this case, the ‘PrK-ONLY’ model’s substandard performance is therefore within anticipation.

#### 4.2.3. Analysis of the Influence of Training Data Size

As discussed in Section 1, large learning datasets are not necessarily available for certain cancer types and may lead to common data-driven failures such as gradient vanishing. However, small datasets can cause insufficient model training, resulting in incomplete models that are not suitable for real-life applications. To mitigate these issues, PrK is often utilised to deal with insufficient learning materials, as the availability of PrK (employed by the KIM) can reduce the underlying classifier’s reliance on training data. We carried out several supporting experiments to verify that a KIM-employed model is not sensitive to a reduced dataset.

**Figure 12:**
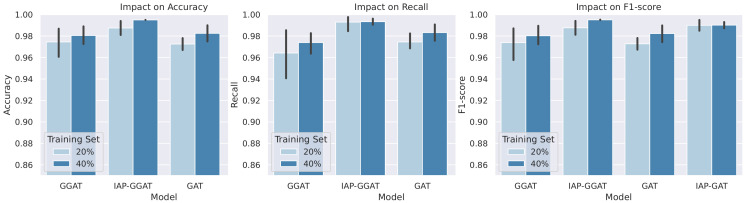
Comparisons amongst models trained on original and the reduced training sets in terms of 3 metrics, i.e., accuracy, recall and F1-score (from left to right). The figures show average model performance (in terms of the corresponding metric) on HNSC and LUSC datasets. Black sticks on top of the bars represent 95% confidence intervals.

We performed a series of additional experiments using the same settings as those in Section 4.2.2, except that we only used HNSC and LUSC datasets, as the model’s performance on them is less diverged (so the resulting statistics could be more accurate), and the scope of candidate models is narrowed down to (IAP-)GAT and (IAP-)GGAT for simplicity. Specifically, we trained each model using two differently sized training sets (20% and 40%). Each model was trained and tested five times with each training set, i.e., each dataset and model pair were trained 10 times in total. We plotted bar charts (Figure 12) for each metric (accuracy, recall and F1-score) to make comparisons and evaluate the performance of the models. The statistical facts (average and standard deviation values) that we used to plot the bar charts are reported in Appendix B.

The charts point out that the IAP-GGAT achieves the highest scores in the three reported metrics. Furthermore, the IAP models (IAP-GGAT, IAP-GAT) all achieve higher scores than the base models (GGAT, GAT) before and after the training data reduction, which reveals the effectiveness of the KIM. The most significant finding in this additional study is that despite tiny effects of reduced datasets can be observed, IAP models can often mitigate the influence, resulting in acceptable scores in certain circumstances where the training data have a limited size.

#### 4.2.4. Analysis of Training Time

**Figure 13:**
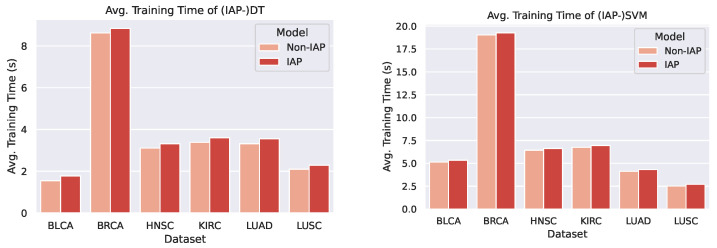
Comparison of (IAP-)DT’s and (IAP-)SVM’s average training time.

**Figure 14:**
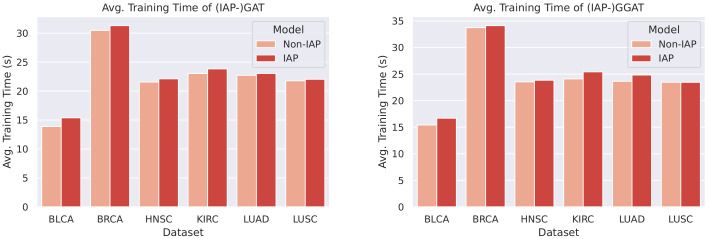
Comparison of (IAP-)GAT’s and (IAP-)GGAT’s average training time.

We plotted bar charts to compare the training time of the IAP and normal models, as shown in Figure 13 and Figure 14.

From the bar chats, we can observe a marginal difference between the average training time of a normal model and its IAP implementation (shorter than 1 s). Despite the small increase in training time, the IAPs achieve significantly better performance than the non-IAPs. Furthermore, the increments in training time on different datasets and of different models are roughly the same (that is, the time complexity caused by the introduction of IAP is O(1)). This means that for even larger datasets, an IAP model could achieve higher performance whilst controlling the increase in its training time within a certain scope.

## 5. Conclusions and Future Work

In this paper, we proposed a novel IML architecture, IAP, that includes the KIM for cancer prediction. This model fuses multiple sources of data to make use of more knowledge available at hand. Unlike the other PrK-based models, we fused the knowledge in a different way that aims at enhancing prediction instead of features, which is more generalisable because the underlying classifier can be specifically designed and fine-tuned for the targeted task.

As an initial attempt, the PrK that we used in this study is both simple and unimodal, though it actually aided the prediction process and truly improved the performance. In our future study, we plan to integrate multimodal PrK (including medical images, gene description texts, etc.) into the learning process, thereby providing sufficient information to enhance our implemented IAP model’s performance to a greater extent. Moreover, we will attempt to verify the interpretability of our architecture according to certain specifications so as to ensure its safety of usage under actual production circumstances.

## Figures and Tables

**Table 1:** List of symbols.

Notation	Description
*N*	The number of samples
$N_{k}$	The number of genes available in the PrK data source
$e_{i}$	The feature vector (embedding) of sample *i*
$k_{c}$	The PrK vector of cancer type *c*
$h_{i}^{\left(l\right)}$	The hidden state of entity *i* in the *l*-th layer
$e_{im}$	The *m*-th component in vector $e_{i}$
$l_{ij}$	The distance between vectors $e_{i}$ and $e_{j}$
A	The adjacency matrix
$A_{ij}$	The component in the *i*-th row and *j*-th column of matrix A
E	The feature matrix

**Table 2:** Statistics of datasets. # Tumour and # Normal indicate the number of samples labelled tumour and normal, respectively. # Edges indicates the number of connections (edges) in the graph that was constructed according to vector distance (see Section 3.3.2).

Dataset	# Tumour	# Normal	# Edges
BLCA	414	19	49,661
BRCA	1102	113	418,792
HNSC	500	44	78,373
KIRC	538	72	109,593
LUAD	533	59	86,536
LUSC	502	49	79,198

**Figure A1:**
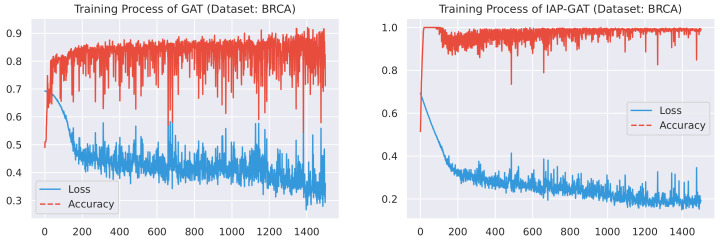
Training process of GAT and IAP-GAT on BRCA dataset.

**Figure A2:**
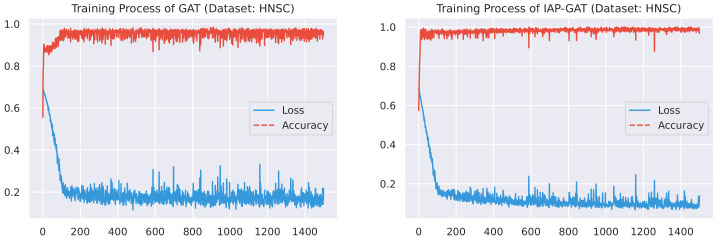
Training process of GAT and IAP-GAT on HNSC dataset.

**Figure A3:**
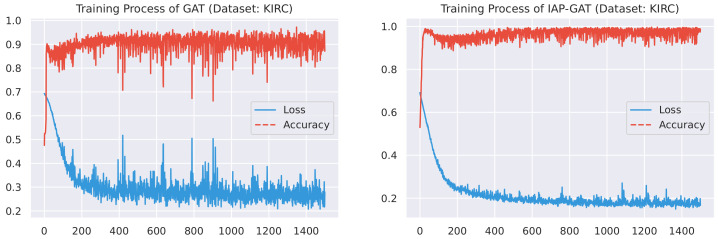
Training process of GAT and IAP-GAT on KIRC dataset.

**Figure A4:**
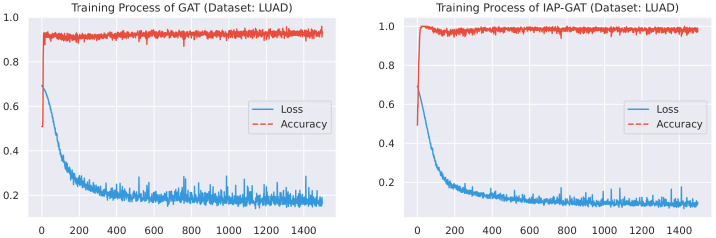
Training process of GAT and IAP-GAT on LUAD dataset.

**Figure A5:**
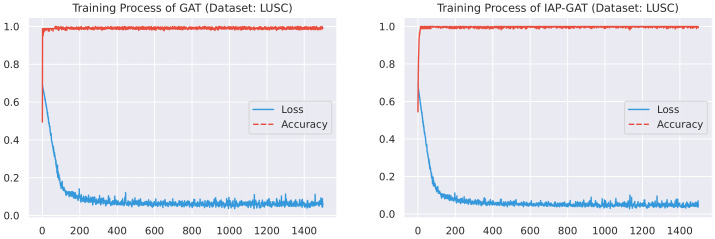
Training process of GAT and IAP-GAT on LUSC dataset.

## Data Availability

The TCGA datasets were downloaded from the NIH GDC database (https://portal.gdc.cancer.gov/ (accessed on 19 September 2021)).

## Appendix A. A Record of Training Process (GAT and IAP-GAT)

In addition to Figure 6, Figure 7, Figure 8, Figure 9, Figure 10 and Figure 11, we plotted the changes in accuracies and loss values during GAT and IAP-GAT’s training on all six datasets. Likewise, a much slighter volatility can be observed on IAP-GAT compared to its non-IAP counterpart, GAT. Despite the notable differences in volatility between (IAP-)GAT and (IAP-)GGAT when reaching their plateaus, the effect of knowledge integration is highlighted here as well.

## Appendix B. Statistical Information

Here, we report some statistical information of Figure 12 that was used whilst analysing the influence of training sets’ sizes (Section 5). Table A1 and Table A2 show average and standard deviation values of the performance recorded in GAT’s and IAP-GAT’s 10 runs on LUSC and HNSC with 20% and 40% training sets.

**Table A1:** Average and standard deviation values of the performance recorded in GAT’s and IAP-GAT’s 10 runs on LUSC and HNSC with 20% and 40% training sets.

	GAT				IAP-GAT			
	20% TS		40% TS		20% TS		40% TS	
	Avg.	Std.	Avg.	Std.	Avg.	Std.	Avg.	Std.
**Accuracy**	0.97255	0.00921	0.98251	0.01251	0.99000	0.00834	0.99001	0.00501
**Recall**	0.97450	0.01118	0.98324	0.01290	0.98937	0.00935	0.98782	0.01171
**F1**	0.97276	0.00895	0.98234	0.01276	0.99000	0.00834	0.99017	0.00481

**Table A2:** Average and standard deviation values of the performance recorded in GGAT’s and IAP-GGAT’s 10 runs on LUSC and HNSC with 20% and 40% training sets.

	GAT				IAP-GAT			
	20% TS		40% TS		20% TS		40% TS	
	Avg.	Std.	Avg.	Std.	Avg.	Std.	Avg.	Std.
**Accuracy**	0.97451	0.02222	0.98052	0.01332	0.98750	0.01084	0.99501	0.00001
**Recall**	0.96420	0.03662	0.97394	0.01620	0.99298	0.01224	0.99340	0.00432
**F1**	0.97389	0.02314	0.98027	0.01426	0.98768	0.01066	0.99511	0.00024
